# Suicidal behaviour in old age - results from the Ibadan study of ageing

**DOI:** 10.1186/1471-244X-13-80

**Published:** 2013-03-13

**Authors:** Akin Ojagbemi, Bibilola Oladeji, Taiwo Abiona, Oye Gureje

**Affiliations:** 1 Department of psychiatry, College of Medicine, University of Ibadan, Ibadan, P.M.B 5017 (GPO), Nigeria; 2 Department of Preventive Medicine and Primary Care, Faculty of Clinical Sciences, College of Medicine, University of Ibadan, Ibadan, P.M.B 5017 (GPO), Nigeria

**Keywords:** Suicide, Suicidal ideation, Aged, Social isolation

## Abstract

**Background:**

An important reason for the high risk of suicide in the elderly is the determination with which they act out their suicidal thoughts. Early identification of suicidal behaviours in the elderly is therefore important for suicide prevention efforts in this population.

**Method:**

Data are from the Ibadan Study of Ageing (ISA), a household multi-stage probability sample of 2149 Yoruba Nigerians aged 65 years or older conducted between 2003 and 2004. We used the third version of the World Health Organization (WHO) Composite International Diagnostic Interview (CIDI) to explore suicidal experiences and behaviours. In this report, only those experiences or behaviours reported to have occurred after the age of 65 years are the focus of analysis. Derived weights were applied to the data in accordance with the study design and associations were explored using logistic regression. The results are presented as odds ratios (ORs) with 95% confidence intervals.

**Result:**

In all, 4.0% (95% C.I= 3.1-4.2) of the subjects had suicidal ideation occurring after the age of 65 years, while 0.7% (95% C.I=0.4-1.3) and 0.2% (95% C.I= 0.1-0.4) reported suicidal plans and attempts, respectively. There was a significantly elevated likelihood of suicidal ideation among persons who had experienced spousal separation through death or divorce (O.R=4.9., 95% C.I= 1.5-15) or who were residing in rural settings (O.R=2.5, 95% C.I=1.3-4.8).

**Conclusion:**

Suicidal ideation is common among the elderly. About 20% and 6% of those with ideation proceed to plans and attempts, respectively. Circumstances of social isolation and exclusion are important correlates of suicidal behaviour in the elderly.

## Background

Suicide is the most common and arguably the most important psychiatric emergency. It is defined by the World Health Organization (WHO) as “the act of killing oneself deliberately initiated and performed by the person concerned in the full knowledge or expectation of its fatal outcome” [1]. It was the cause of 1.8% of the world’s 54 million deaths in 1998 [2], and an estimated 1 million death in the year 2000 [3]. Over 10 times the number of completed suicides is often reported for attempts. The worldwide incidence of suicide increases with age, with the rate of suicide in those aged over 75 years reaching up to twice or three times the rate in those younger than 25 years in most countries [4-6]. Therefore, older adults are at a substantial risk of dying by suicide compared to the younger age categories. As reported by the WHO for 10 countries and a report from South-India, this pattern may be more common in developing countries [7,8]. The proportion of completed suicide among suicide attempters is also higher in this age category, with 1 suicide reported for every 4 attempts in older adults compared to about 1 completed suicide out of 12 attempts in the general population [9]. For these reasons, suicidal behaviour in the elderly is a major public health concern.

Suicide is a complex phenomenon. It is difficult to predict precisely, and has numerous associated factors which may be biological, psychosocial or an inter-play of these factors [9]. But suicidal ideation, suicidal attempts and actual suicide frequently occur on a continuum. Thus, suicidal thoughts are frequently the first step in the process of eventual suicide [10,11], and the elderly who has suicidal ideation may be more likely to act out their suicidal intentions through more determined and lethal techniques [12]. Therefore suicidal behaviours such as ideation and plans have variously been considered as pointers to imminent suicide [13]. Elderly persons with suicidal intention are less likely to communicate this to people around them [14]. Even when intentions have been communicated, there is evidence that such are often difficult to understand or to deal with [15]. Epidemiological studies may provide useful information about the predictors of suicidal behaviours and the transition from one behaviour to another. Such studies are yet to be conducted among elderly persons living in sub-Saharan Africa. We report on a large community-based study of suicidal behaviour among elderly persons in Nigeria.

## Method

The Ibadan Study of Aging (ISA) is a community-based longitudinal survey of the mental and physical health status of elderly persons (aged 65 years and over). Its methodology has been described in full elsewhere [16,17] and only a brief account is provided here. The study was conducted in the Yoruba-speaking areas of Nigeria, consisting of eight contiguous states in the south-western and north-central regions (Lagos, Ogun, Osun, Oyo, Ondo, Ekiti, Kogi and Kwara). These states account for about 22% of the Nigerian population (approximately, 25 million people). The baseline survey, on which the present report is based, was conducted between November 2003 and August 2004.

### Sample

We selected one respondent per household using a multi-stage stratified area probability sampling of households. When more than one elderly person was eligible for study, (eligibility criteria were being aged 65 years or older and fluent in the language of the study, Yoruba), the Kish table selection method was used to select the one respondent [18]. Respondents were informed about the study, invited to participate, but also assured of their right to decline. Participants were those who provided consent, mostly verbal, either because of illiteracy or by choice, before interviews were conducted. On the basis of this selection procedure, face-to face interviews were carried out on 2152 respondents (of which 2149 were complete), giving a response rate of 74.2%. Non-response was predominantly due to non-availability after repeated visits (14%), interviewers unable to trace the original address (4%), death (3%), and physical incapacitation (2%) and rarely due to refusal (2%).

The survey was approved by the University of Ibadan/University College Hospital, Ibadan Joint Ethical Review Board.

### Measures

Suicidal behaviour was assessed with the 3^rd^ version of the Composite International Diagnostic Interview (CIDI 3.0) [19]. The CIDI first lists three possible experiences: suicidal ideas (described as the presence of serious thoughts of committing suicide), plans (described as a report of a serious plan of committing suicide) and attempts (described as the occurrence of a serious attempt at committing suicide). The interview first explores the lifetime experience of each outcome and, if the subject answers in the affirmative, questions are then asked about the age of the subject at the first ever experience, the last experience and whether the respondent currently has the experience. The subject is further asked to state the number of times each event has ever occurred and if suicidal attempt resulted in some injury or needed medical attention. If the subject admits to having made suicide attempt, the interviewer asks whether the attempt was really serious and the subject survived only as a matter of luck or the subject either simply wanted to attract attention or used a suicide method knowing it was not fool-proof. Only suicidal experiences or behaviours that occurred after the age of 65 are included in this report.

#### Depression

Depression was assessed with the depression module of the CIDI. Diagnosis was based on the criteria of the Diagnostic and Statistical Manual of Mental Disorders, Fourth Edition (DSM-IV) [20]. DSM-IV organic exclusion rules were imposed in making the diagnosis of depression.

### Social network

Social network was assessed with the CIDI. The relevant items enquire about the frequency of respondent’s contact with family members who do not live with the respondent and frequency of contact with friends. In this report, we have dichotomize the responses to contacts that are less than once in six months to those more than once in six months.

### Chronic physical health conditions

#### Chronic health conditions

A checklist of chronic physical and pain conditions was included in the ISA. Respondents were asked if they had any chronic respiratory conditions (asthma, tuberculosis, other lung disease), cardiovascular conditions (high blood pressure, heart disease, heart attack, stroke), cancer, diabetes, or epilepsy. Respondents were asked whether they had experienced each of the symptom-based conditions in the previous 12 months. The checklist also ascertained the presence of any chronic pain. These included back or neck pain, arthritis, frequent headaches, and a general category of chronic pain in any other body parts.

All the instruments used in the ISA were translated using iterative back-translation method. As part of the translation process, all the instruments used were subjected to cultural adaptation. Thus, for example, the list of household items included items common in the culture.

### Data analysis

The prevalence of each of the three suicidal behaviours, ideation, plan and attempt, is presented. Associations with socio-economic variables were explored using logistic regression and the results are presented as odds ratios (ORs) with 95% confidence intervals. We used a validated method of estimating economic status of elderly persons in low income settings by obtaining information on the possession of 21 household and personal items such as chairs, clock, bucket, radio, television set, fans, stove or cooker, car, telephone, etc. [21]. We next categorized each respondent’s economic status by relating their total possessions to the median number of possessions of the entire sample. The resulting categories are low for a ratio 0.5 or less to the median, low-average for a ratio of 0.5 – 1.0, high-average for 1.0 – 2.0, and high if the ratio is over 2.0. Residence was classified as rural (less than 12,000 households), semi-urban (12,000 – 20,000 households) and urban (greater than 20,000 households).

The analysis took account of the stratified multistage sampling procedure and the associated clustering by applying weights as appropriate. We made adjustment for differences between the sample and the total Nigerian population by applying post-stratification to the target sex and age range. The weight so derived was normalized to reset the sum of weights back to the original sample size of 2149. To take account of the complex sample design and weighting, we used the jacknife replication method implemented with the STATA statistical package to estimate standard errors for proportions [22]. Predictor factors for each suicidal behaviour were explored with logistic regression analysis [23] and the results are presented as odds ratios (OR’s) with 95% confidence intervals (CI’s), and all the CI’s are adjusted for design effects. All analyses were conducted with the STATA statistical package.

## Results

### Characteristics of subjects

There were 992 males (46.2%) and 1157 (53.8%) females in the study sample. The mean age of the cohort was 75.06 ± 9.2 years. Men were significantly younger with a mean age of 74.27 ± 9.2 years, compared to a mean age of 75.74 ± 9.2 years for women (p < 0.001). One thousand one hundred and eighty four subjects (55.1%) had no formal education, while 533 (24.8%) had up to 6 years of formal education and the remaining 432 (20.1%) subjects had more than 6 years of formal education. Primary school education lasts for 6 years in Nigeria and this is expected to make one literate. Majority of the subjects, 2,095 (97.5%), had a religious affiliation. One thousand four hundred and twenty five (66.3%) subjects lived in urban or semi-urban locations, while the remaining 724 (33.7%) were domiciled in rural locations. One thousand and eighty subjects (50.3%) were currently in a marital relationship, while 1069 (49.7%) were not, with almost all being widowed. One thousand five hundred and fifty three (72.3%) subjects lived in houses with mud floor, while the remaining 596 (27.7%) lived in houses with a hard floor design. A lifetime diagnosis of depression was made in 551 (25.6%) of the subjects in the study sample. The distribution of the economic groups as well as other characteristics of the subjects are shown in Table 1.

**Table 1:** Characteristics of subjects

**Characteristics**	**Number**	**Percentage**
**Age group** (years)		
80+	657	30.6
75–79	295	13.7
70–74	495	23.0
65–69	702	32.7
**Mean** (**SD**)	**75.06** (**9**.**2**)	
**Gender**		
Male	992	46.2
Females	1157	53.8
**Years of formal education**		
0	1184	55.1
1–6	533	24.8
>6	432	20.1
**Marital group**		
Married	1080	50.3
Separated/Widowed/Divorced	1069	49.7
**Economic group**		
(High)	224	10.4
(High-average)	495	23.0
(Low-average)	763	35.5
(Low)	667	31.0
**Residence**		
Urban	555	25.8
Semi-urban	870	40.5
Rural	724	33.7
**Quality of house floor**		
Earth floor	1553	72.3
Hard floor	596	27.7
**Religious**	2095	97.5
**Contact with family**	2054	95.6
**Contact with friends**	1809	84.2
**Lifetime depression**	551	25.6
**Presence of chronic medical conditions**	628	29.2
**Presence of chronic pain**	1700	79.1

### Frequency of suicidal behaviour

In all, 99 (4.0%) subjects had experienced suicidal ideation, while 20 (0.7%) of them reported a history of suicidal plans. Six (0.2%) subjects reported a previous attempt at suicide.

Tables 2 and 3 show the comparison of the characteristics of subjects with the 3 main suicidal behaviours: ideation, plan, and attempt. There was a linear relationship between residence and risk of suicidal ideation: compared to respondents residing in urban areas, those in semi-urban areas had a (non-significant) increase in risk while there was an almost 3-fold increase in risk for respondents residing in rural areas (Table 3). The associations between suicidal behaviour and age, sex or level of education were not significant. Similarly, the relationship between suicidal behaviour and economic status was not significant. However subjects who were ever, but no longer, married, mainly through death of their spouses, were about 5 times more likely to have suicidal plans compared to those who remained married (O.R=4.9., 95% C.I= 1.5-15) (Table 3).

**Table 2:** The distribution of suicidal behaviour according to the characteristic of subjects

**Characteristics**	**Ideation n. (%)**	**Plan. n (%)**	**Attempt n (%)**
**Age group** (years)			
80+	32 (4.3)	11 (1.4)	3 (0.5)
75–79	17 (4.7)	1 (0.5)	0.(0.0)
70–74	28 (4.7)	4 (0.6)	2 (0.2)
65–69	22 (3.0)	4 (90.7)	1 (0.1)
**Gender**			
Male	39 (0.7)	2 (0.2)	1 (0.1)
Females	60 (4.5)	18 (1.5)*	5 (0.3)
Total	99 (4.0)	20 (0.7)	6 (0.2)
**Years of formal education**			
0 1–6	51 (3.9)	11 (0.8)	2 (0.1)
7–12	29 (4.4)	4 (0.7)	2 (0.3)
13+	10 (3.8)	3 (1.2)	1 (0.2)
	9 (4.0)	2 (0.2)	1 (0.1)
**Marital group**			
Married	44 (3.4)	5 (0.3)	3 (0.1)
Separated/Widowed/divorced	55 (5.0)	15 (1.5)**	3 (0.3)
**Economic group**			
High	8 (3.6)	2 (0.3)	1 (0.3)
High-average	22 (3.8)	3 (0.6)	0 (0.0)
Low-average	43 (4.6)	10 (0.9)	5 (0.4)
Low	26 (3.6)	5 (0.9)	0 (0.0)
**Residence**			
Urban	16 (2.5)	5 (0.8)	2 (0.2)
Semi-urban	41 (3.4)	9 (0.8)	3 (0.2)
Rural	42 (6.1)**	6 (0.7)	1 (0.1)
**Quality of house floor**			
Earth floor	74 (4.2)	17 (1.0)	5 (0.2)
Hard floor	25 (3.6)	3 (0.3)	1 (0.1)
**Religious**	97 (4.0)	20 (0.8)	6 (0.2)
**Contact with family**	96 (4.0)	19 (0.7)	6 (0.2)
**Contact with friends**	89 (4.2)	18 (0.7)	6 (0.2)
**Lifetime depression**	551	25.6	1 (0.2)

*= p<0.05, ** = p<0.01.

**Table 3:** Predictors of suicidal behaviour among subjects

**Characteristics**	**Ideation (n=99) O.R (95% C.I)**	**Plan (n=20) O.R 56789=(95% C.I)**	**Attempt (n=6) O.R (95% C.I)**
**Age group** (years)			
80+	1	1	1
75–79	1.1 (0.6–2.2)	0.3 (0.0–3.2)	-(−)
70–74	1.1 (0.7–1.9)	0.4 (0.1–1.6)	0.4 (0.1–2.7)
65–69	0.7 (0.4–1.3)	0.5 90.1–1.7)	0.2 (0.1–2.1)
**Gender**			
Male	1	1	1
Females	0.8 (0.5–1.5)	0.2 (0.0–0.8)	0.2 (0.0–2.5)
**Marital group**			
Married	1	1	1
Separated/Widowed/Divorced	1.5 (0.9–2.5)	4.9 (1.5–15.7)	2.6 (0.5–14.5)
**Years of formal education**			
13+	1	1	1
7–12	1.0 (0.5–2.0)	5.3 (0.7–39.1)	1.6 (0.1–28.3)
1–6	1.1 (0.4–3.0)	3.0 (0.4–22.10	1.9 (0.2–22.4)
0	1.0 (0.5–2.1)	3.5 (0.4–28.6)	0.8 (0.1–10.1)
**Economic group**			
High	1	1	1
High-average	1.1 (0.4-3.1)	1.5 (0.2-12.7)	-(−)
Low-average	1.3 (0.5-3.5)	2.4 (0.4-14.8)	1.4 (−)
Low	1.0 (0.4-2.6)	2.5 (0.4-15.3)	-(−)
**Residence**			
Urban	1	1	1
Semi-urban	1.3 (0.7–2.7)	1.0 (0.3–3.9)	0.8 (0.0–5.7)
Rural	2.5 (1.3–4.8)	1.0 (0.2–-4.9)	0.5 (0.0–5.6)
**Quality of house floor**			
Earth floor	1	1	1
Hard floor	0.9 (0.5–1.5)	0.3 (0.1–1.1)	0.5 (0.1–4.9)
**Contact with family**			
**Yes**	1	1	1
**No**	0.8 (0.3–2.1)	4.1 (0.6–30.7)	-(−)
**Religious**			
yes	1	1	1
No	0.7 (0.2–2.8)	-(−)	-(−)
**Contact with friends**			
Yes	1	1	1
No	0.6 (0.3–1.2)	1.2 (0.2–7.2)	-(−)
**Lifetime depression**			
**Yes**	1.1 (0.5–2.2)	1.6 (0.5–4.8)	0.8 (0.1–7.9)
**No**	1	1	1

## Discussion

To our knowledge this is the first report of suicidal behaviour among community dwelling elderly persons in sub-Saharan Africa. We sought to identify the prevalence of three behaviours which are known to occur in a continuum between normality and actual suicide. In this study about 1 in 20 elderly persons had experienced suicidal thoughts since they attained the age of 65 years while about 1 in 100 had made suicidal plans during this period. Given that the mean age of the sample at assessment was about 75 years, the suggestion is that these events had occurred over a period of about 10 years. The three reported behaviours provided a pattern that is not uncommon: thus while about 20% of persons with ideation in old age had made a plan, the proportion who had made an attempt among ideators during the period was 6%. A somewhat similar pattern has also been reported in an earlier study in the general population of Nigerians above the age of 18 years [24].

Estimates of suicidal ideation vary widely across studies depending on the methodology of the study, the time frame considered, or the comprehensiveness of the assessment instrument. Thus, studies of community dwelling older adults from other parts of the world report that suicidal ideation occur at rates that range between 2.16% and 17% [25]. Direct comparison with other large surveys is difficult since our assessment of suicidal behaviour was specifically made on the period since the respondents attained the age of 65 years, rather than over the entire lifetime. Also, we cannot be sure of the extent to which reluctance to report these behaviours might have affected our rates. Suicidal behaviours still carry a significant level of stigma in the cultural setting of our study. However, our results show a trend similar to that reported among community dwelling older people in previous studies conducted in the West [26,27]. However, we note that suicidal ideation in this elderly sample is much higher than in the general Nigerian adult population in which a figure of 3.2% has been reported over the lifetime in a community sample with a mean age of about 35 years [24]. This finding suggests that old age, at least in the country setting of our study, is a significantly stressful period of life and a risk period for suicidal behaviour. The observation is in consonance with a previous report from our group suggesting one of the highest rates of major depression, both current and lifetime, among elderly persons anywhere [17]. Although most people with suicidal ideation will not progress to suicidal attempt, the older adult is however more likely to make determined effort at progressing through to actual suicide using the most lethal methods compared to younger subjects [12]. The fact that only 0.2% of our sample admitted to have made a lifetime suicidal attempt does not contradict this evidence. Since we do not have information about those who might have committed suicide in old age, the possibility exists that perhaps our observation only reflects the fact that only those who survived an attempt were alive to tell the story of their attempt. Furthermore, cultural issues about admitting to previous suicidal attempts may have affected the rates of attempted suicide reported in this study.

We also sought to identify the predictors of suicidal behaviours in this population. Living in a rural location predicted the occurrence of suicidal ideation, while being separated either through death or divorce predicted suicidal planning. In the period before the actual suicide, older people are known to experience various kinds of losses that heavily burden them. The loss of a partner has been particularly linked to an increased risk of suicide, especially in men [28-30]. Spousal loss reduces the amount of social support available to the elderly and thus increases the likelihood of social isolation. It has been argued that increased likelihood of social isolation among the elderly compared to younger persons is one of the reasons for the greater risk of suicide in the former [31]. Social isolation also predisposes to depression, a major risk factor for suicide [27,30]. Social isolation as well as exclusion may also explain why, in this study, living in a rural location predicted suicidal ideation. Although it has previously been argued that social isolation is more common in large populations [32], the circumstances leading to social isolation in the elderly in contemporary Africa may be slightly different. For instance, in Nigeria as in many low income countries, the young and able bodied individual drift from rural areas to urban centres seeking better economic opportunities, leaving behind a population of older persons in the rural areas. The result is a reduction of the social network available to older persons. Furthermore, in Nigeria as in other sub-Saharan countries going through social changes, there is a gradual but continuous erosion of the traditional extended family system. Typically, rural areas are deprived of social amenities and opportunities for recreation. When social amenities are available, they are often not tailored to the needs of the older person. The pattern of the relationship between rural dwelling and suicidality has been reported in the general population of many countries [33,34]. This appears to be in keeping with the theory of Durkheim in which poor social integration leads to increased suicide in the population [7]. Depression has often been reported in the literature as the most important predictor of suicidal behaviour in older adults, especially given its relationship with other risk factors such as social isolation, disability and illness [27,30,35]. However lifetime depression was not a significant predictor of suicidal behaviour in this study. One reason for this could be due to the use of different timeframes for the two measures: lifetime for depression and old age for suicidal behaviour. Perhaps a different pattern might have been observed if depression since age 65 years was examined. The possibility that recall bias might have affected the assessment of lifetime depression is also a plausible factor. Also, because of the cross-sectional nature of the data, we were unable to clearly differentiate depressive episodes occurring before suicidal behaviour, the variable of interest, from those occurring after. The effect of this would be to introduce a lot of “noise” to the examination of the association with the resultant loss of statistical power. For these reasons, we could not rule out the potential role of depression in predicting suicidality in our sample. Nevertheless, some similarly designed studies have reported that only a minority of persons with suicidal thoughts have depression symptoms sufficient for the diagnosis of a major depressive disorder even though they may have depressed mood and some other associated symptoms [11,36]. This finding reflects the importance of several other variables apart from depression in the complex process of suicidality.

In interpreting the results of our study, it is important to bear in mind its limitations. These include its reliance on a retrospective measure of suicidality and depression, as well as the cross-sectional nature of the study. As noted earlier, stigma around the topic of suicide is a potent factor in the population in which our study was conducted and may have resulted in conservative estimates of suicidal behaviour. Nevertheless, the findings are novel in so far as they present the picture of an important public health issue in this under-studied population.

## Conclusions

Suicidal ideation is common in the elderly, and it would appear from this study that suicidality in this age group occurs in the same spectrum of continuity that has been reported in other age categories, where suicidal ideations are the first step towards eventual suicide. Life events and other factors that lead to e social isolation in the elderly are important predictors of suicidal behaviour in this population. Suicide prevention efforts should include provision of opportunities for participation of the elderly in social and recreational activities in their communities.

## Competing interests

None of the authors have a competing interest.

## Authors’ contributions

AA produced the first draft of the manuscript, revised the final draft and approved it for publication, BO was involved in the conception of this work, revised the final draft of the manuscript and approved for publication, TA carried out the statistical analyses and approved for publication, and OG conceived the work, procured funds, produced the first draft, revised the final draft and approved for publication.

## Pre-publication history

The pre-publication history for this paper can be accessed here:

http://www.biomedcentral.com/1471-244X/13/80/prepub
